# Mitochondrial complex I inhibition triggers a mitophagy-dependent ROS increase leading to necroptosis and ferroptosis in melanoma cells

**DOI:** 10.1038/cddis.2017.133

**Published:** 2017-03-30

**Authors:** Farhan Basit, Lisanne MPE van Oppen, Laura Schöckel, Hasse M Bossenbroek, Sjenet E van Emst-de Vries, Johannes CW Hermeling, Sander Grefte, Charlotte Kopitz, Melanie Heroult, Peter HGM Willems, Werner JH Koopman

**Affiliations:** 1 Department of Biochemistry, Radboud Institute for Molecular Life Sciences (RIMLS), Radboud University Medical Centre (Radboudumc), Nijmegen, The Netherlands; 2 Global Therapeutic Research Group Oncology II, Bayer Pharma AG, Berlin, Germany; 3 Bayer AG Innovation Strategy, Leverkusen, Germany

## Abstract

Inhibition of complex I (CI) of the mitochondrial respiratory chain by BAY 87-2243 (‘BAY') triggers death of BRAF^V600E^ melanoma cell lines and inhibits *in vivo* tumor growth. Here we studied the mechanism by which this inhibition induces melanoma cell death. BAY treatment depolarized the mitochondrial membrane potential (Δ*ψ*), increased cellular ROS levels, stimulated lipid peroxidation and reduced glutathione levels. These effects were paralleled by increased opening of the mitochondrial permeability transition pore (mPTP) and stimulation of autophagosome formation and mitophagy. BAY-induced cell death was not due to glucose shortage and inhibited by the antioxidant *α*-tocopherol and the mPTP inhibitor cyclosporin A. Tumor necrosis factor receptor-associated protein 1 (TRAP1) overexpression in BAY-treated cells lowered ROS levels and inhibited mPTP opening and cell death, whereas the latter was potentiated by TRAP1 knockdown. Knockdown of autophagy-related 5 (ATG5) inhibited the BAY-stimulated autophagosome formation, cellular ROS increase and cell death. Knockdown of phosphatase and tensin homolog-induced putative kinase 1 (PINK1) inhibited the BAY-induced Δ*ψ* depolarization, mitophagy stimulation, ROS increase and cell death. Dynamin-related protein 1 (Drp1) knockdown induced mitochondrial filamentation and inhibited BAY-induced cell death. The latter was insensitive to the pancaspase inhibitor z-VAD-FMK, but reduced by necroptosis inhibitors (necrostatin-1, necrostatin-1s)) and knockdown of key necroptosis proteins (receptor-interacting serine/threonine-protein kinase 1 (RIPK1) and mixed lineage kinase domain-like (MLKL)). BAY-induced cell death was also reduced by the ferroptosis inhibitor ferrostatin-1 and overexpression of the ferroptosis-inhibiting protein glutathione peroxidase 4 (GPX4). This overexpression also inhibited the BAY-induced ROS increase and lipid peroxidation. Conversely, GPX4 knockdown potentiated BAY-induced cell death. We propose a chain of events in which: (i) CI inhibition induces mPTP opening and Δ*ψ* depolarization, that (ii) stimulate autophagosome formation, mitophagy and an associated ROS increase, leading to (iii) activation of combined necroptotic/ferroptotic cell death.

To sustain their function and proliferation melanoma cells often shift their metabolism from mitochondrial towards glycolytic ATP production.^1^ However, various oncogenes and tumor suppressors (e.g. c-myc, Ras and Oct1), as well as hypoxia, stimulate mitochondrial metabolism.^2, 3, 4, 5^ A key oncogenic event in melanoma is the occurrence of mutations in v-Raf murine sarcoma viral oncogene homolog B (BRAF). This protein kinase is involved in RAS–RAF–MEK–ERK mitogen-activated protein kinase signaling.^1^ Among the BRAF mutations, the V600E gain-of-function substitution is most commonly observed (i.e. in 40–60% of all melanomas). In addition, BRAF mutations were demonstrated in 40% of the thyroid cancers, 30% of the ovarian cancers and 20% of the colorectal cancers.^6, 7^ Despite novel antitumour therapeutics, metastatic melanoma still has a poor prognosis due to the development of chemotherapy resistance.^8^ Importantly, acquired resistance to BRAF or MEK inhibitors was paralleled by increased mitochondrial biogenesis and activity in melanoma cells with BRAF^V600E^ and NRAS mutations.^9, 10^ This suggests that concomitant inhibition of mitochondrial function might constitute a potential therapeutic strategy.^11, 12^ Proper mitochondrial functioning requires activity of the mitochondrial oxidative phosphorylation (OXPHOS) system.^13, 14, 15^ This system is embedded in the mitochondrial inner membrane (MIM) and consists of four electron transport chain (ETC) complexes (CI–CIV) and the F_0_F_1_-ATP-synthase (CV). OXPHOS generates ATP through chemiosmotic coupling by linking ETC-mediated proton efflux across the MIM to CV-mediated trans-MIM proton influx.^16^ The latter is driven by the inward-directed proton motive force across the MIM, which consists of an electrical (Δ*ψ*) and chemical (ΔpH) component, with Δ*ψ* contributing ~85% to the total PMF.^17^

Using a panel of BRAF^V600E^ melanoma cell lines, we recently demonstrated that BAY 87-2243 (BAY; Ellinghaus *et al.*^18^), a potent inhibitor of the first OXPHOS complex (CI or NADH:ubiquinone oxidoreductase; EC 1.6.5.3), dose-dependently decreases cell viability.^19^ The effect of BAY was paralleled by increased levels of cellular ROS and a reduction in cellular ATP levels. Measurements were performed using a medium with a physiological glucose concentration (5 mM) and cell death was prevented by increasing this concentration to 25 mM. Remarkably, using the latter concentration also inhibited the BAY-induced ROS increase and reduction in ATP level.^19^ Mechanistically, we hypothesized that BAY-induced CI inhibition promotes cell death by stimulating ATP-depletion and/or ROS generation and subsequent oxidative stress induction. Here we dissected the mechanism by which BAY kills BRAF^V600E^ melanoma cells. Our results suggest that this death is not due to glucose shortage but involves a chain of events by which BAY stimulates opening of the mitochondrial permeability transition pore (mPTP) and Δ*ψ* depolarization, followed by autophagosome formation, mitophagy, a cytosolic ROS increase and combined necroptosis/ferroptosis.

## Results

### BAY treatment induces cell death in BRAF^V600E^ melanoma cell lines

**Figure 1:**
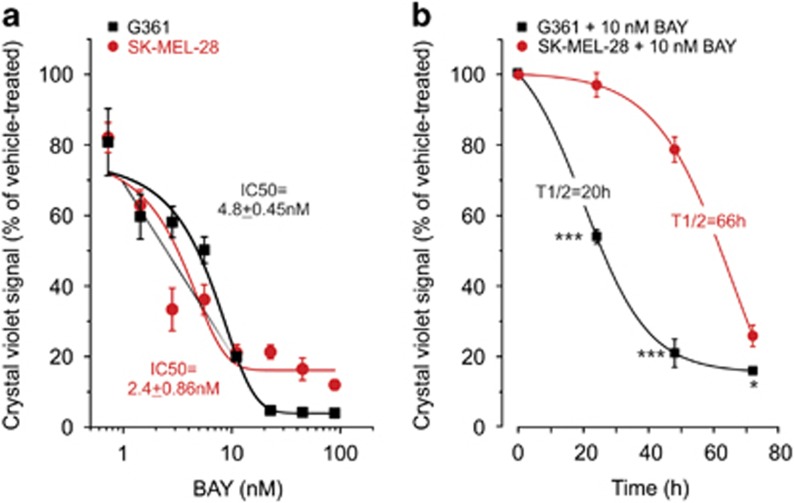
Dose- and time-dependent effect of BAY and medium refreshment on cell viability. (**a**) Dose-dependent effect of BAY (*N*=2, *n*=4) on the viability of G361 and SK-MEL-28 cells (at 72 h). A Boltzmann equation was used to determine the IC_50_ (*x*_0_) value: *y*=(*A*_2_+(*A*_1_−*A*_2_)/(1+exp((*x*−*x*_0_)/d*x*))). (**b**) G361 and SK-MEL-28 melanoma cells were treated with 10 nM BAY (*N*=3, *n*=6) and their viability was measured at different time points. A sigmoidal (logistic) equation was used to determine the *T*_1/2_ (*x*_0_) value: *y*=([(*A*_1_−*A*_2_)/(1+(*x/x*_0_)]+*A*_2_). Statistics: In panel b, significant differences with the SK-MEL-28 cell line are indicated by *P<0.05 and ***P<0.001

**Figure 2:**
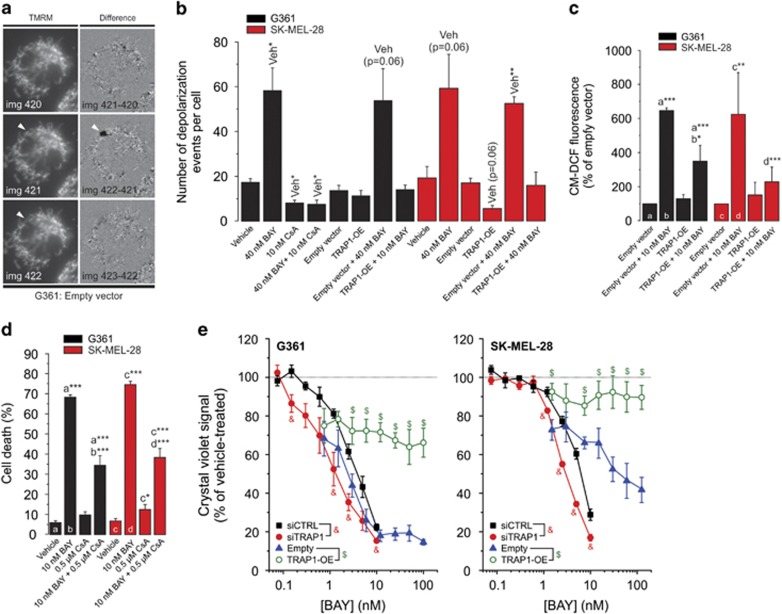
Effect of TRAP1 knockdown/overexpression on the BAY-induced reduction in cell viability. (**a**) Detection of photoinduced mitochondrial membrane potential (Δ*ψ*) depolarizations (‘Δ*ψ*-flickering') in a typical TMRM-stained G361 cells (see Results for details). (**b**) Effect of BAY (2 min), CsA (2 h), empty vector and TRAP1-OE on Δ*ψ*-flickering (*N*=3, *n*≥20). (**c**) Effect of BAY on reactive oxygen species (ROS) levels (at 24 h; N=3, *n*=3) in cells transfected with the empty or TRAP1-OE vector. (**d**) Effect of vehicle (*N*=5, *n*=15), BAY (*N*=5, *n*=15) and CsA (*N*=3, *n*=6) on cell death (G361: at 48 h; SK-MEL-28: at 72 h). (**e**) Effect of BAY on the viability of cells (G361: at 48 h; SK-MEL-28: at 72 h; *N*=3, *n*=6) transfected with siCTRL, siTRAP1, empty vector and TRAP1-OE. Statistics: Significant differences relative to the vehicle condition (‘Veh' in panel b) and between treatments (a, b, c, d in panels **c** and **d**) are indicated by *P<0.05, **P<0.01 and ***P<0.001. In panel (**e**), significant differences (*P*<0.05) between conditions are marked by symbols (&, $)

In this study, we used two BRAF^V600E^ melanoma cell lines (G361 and SK-MEL-28) to investigate the mechanism of BAY-induced cell death. We previously demonstrated^19^ that BAY treatment for 72 h reduced the viability of these cells in a dose-dependent manner with IC_50_ values in the nanomolar range (Figure 1a). Within this timeframe, BAY did not affect the viability of human epidermal melanocytes (Hema-LP) and primary human skin fibroblasts (CT5120; Supplementary Figure S1A). Experiments were performed at an ambient glucose concentration of 5 mM. Importantly, regular refreshment of the culture medium did not prevent the BAY-induced reduction in cell viability, arguing against glucose depletion being responsible for this reduction (Supplementary Figure S1B). In agreement with our previous study,^19^ it was found that BAY displayed a half-maximal inhibition of cell viability (*T*_1/2_) at 20 and 66 h for G361 and SK-MEL-28 cells, respectively (Figure 1b). This means that G361 cells are more sensitive to CI inhibition than SK-MEL-28 cells.^19^ Therefore, to study cell death an incubation time was chosen at which a similar reduction in viability for G361 (48 h) and SK-MEL-28 (72 h) cells was observed. Under these conditions, BAY treatment induced cell death to the same extent (70%) in both cell lines (e.g. Figure 2d).

### TRAP1 overexpression inhibits BAY-stimulated mPTP opening, cellular ROS increase and cell death

Activation of BRAF signalling leads to increased phosphorylation of tumor necrosis factor receptor-associated protein 1 (TRAP1), which displays antiapoptotic properties in cancer cells.^20^ TRAP1 is also involved in the regulation of mPTP opening.^21^ To assess whether TRAP1 and mPTP opening play a role in BAY-induced cell death, we used a previously described method to measure mPTP opening.^22^ In brief, cells are stained with the fluorescent cation tetramethylrhodamine methyl ester (TMRM), which accumulates in the mitochondrial matrix in a Δ*ψ*-dependent manner.^23^ Next, reversible mPTP openings are photoinduced by controlled illumination of TMRM (Supplementary Figure S2 and Supplementary Movie). For quantification, individual mPTP openings were manually counted from ‘difference images' calculated by subtracting the (*n*−1)^th^ image from the *n*^th^ image in the recorded TMRM image sequence (Figure 2a; e.g. img422−img421). In these difference images individual mPTP openings are highlighted in black (Figure 2a; arrowhead). Acute BAY treatment (2 min) increased the number of mPTP openings to a similar extent in both cell lines (Figure 2b). Of note, given the short incubation time we here used a 40 nM BAY concentration. This was the lowest BAY concentration that maximally inhibited oxygen consumption in G361 and SK-MEL-28 cells during acute treatment.^19^ The acute effect of BAY on mPTP opening (Figure 2b) and its chronic effect on cell death (Figure 2d) were inhibited by pre-treatment (2 h) with the mPTP inhibitor cyclosporin A (CsA). TRAP1 overexpression (Supplementary Figure S3A) mimicked both effects of CsA (Figures 2b–e). These results suggest that TRAP1 inhibits mPTP opening to prevent cell death in BRAF^V600E^ melanoma cells and that TRAP1 overexpression is required to inhibit BAY-induced mPTP opening and cell death. In agreement with this idea, TRAP1 knockdown (Supplementary Figure S3A) increased the sensitivity to BAY-induced cell death as compared with siCTRL (Figure 2e). TRAP1 overexpression significantly reduced the increase in cellular ROS levels after 24 h of BAY treatment (Figure 2c). Taken together, these results suggest that stimulation of mPTP opening is a key component of BAY-induced cell death and that endogenous TRAP1 levels and/or TRAP1 activity are too low to protect against this death. The fact that TRAP1 overexpression lowers ROS levels in BAY-treated cells suggests that TRAP1 directly reduces these levels or that mPTP opening induces ROS.

### ATG5 knockdown inhibits BAY-stimulated autophagosome formation, ROS increase and cell death

**Figure 3:**
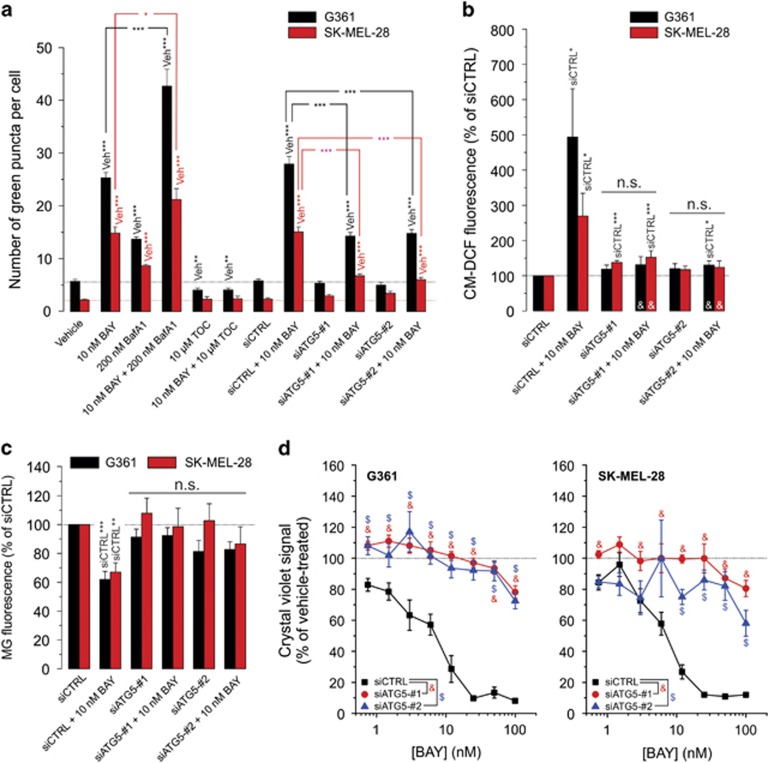
Effect of ATG5 knockdown on the BAY-induced stimulation of autophagy, reactive oxygen species (ROS) increase and reduction in cell viability. (**a**) Effect of BAY in the absence and presence of BafA1, TOC and ATG5 knockdown on the number of green puncta in G361 and SK-MEL-28 cells (at 24 h; *N*=3, *n*=30). (**b**) Effect of BAY on ROS levels (at 24 h; *N*=3, *n*=9) in cells transfected with an empty or GPX4 overexpression vector (GPX-OE). (**c**) Effect of BAY on cellular MG fluorescence (G361: at 24 h; SK-MEL-28: at 24 h; *N*=3, *n*=9) in cells transfected with siCTRL, siATG5-no. 1 and siATG5-no. 2. (**d**) Effect of BAY on the viability of cells (G361: at 48 h; SK-MEL-28: at 72 h; *N*=3, *n*=6) transfected with siCTRL, siATG5-no. 1 and siATG5-no. 2. Statistics: Significant differences relative to the marked conditions are indicated by *P<0.05, **P<0.01 and ***P<0.001. NS indicates nonsignificant. In panel (**d**), significant differences with the (siCTRL+10 nM BAY condition) are marked by ‘&'. In panel (**b**) significant differences (*P*<0.05) between conditions are marked by symbols (&, $)

Elevated ROS levels and increased mPTP opening are established stimulators of autophagy.^24^ To test the hypothesis that BAY stimulates autophagosome formation, cells were transfected with the biosensor mCherry-GFP-tagged LC3. Quantification of the number of green GFP puncta per cell, representing autophagosomes, revealed a significant BAY-induced increase (Supplementary Figure S4A and B and Figure 3a). Co-incubation with bafilomycin A1 (BafA1), which prevents fusion of autophagosomes with lysosomes,^25^ further increased the number of green GFP puncta. This suggests that BAY does not inhibit autophagosome fusion but stimulates autophagosome formation. We previously demonstrated that the antioxidant *α*-tocopherol (TOC) inhibits the BAY-induced increase in cellular ROS levels and cell death.^19^ Here we observed that 24 h cotreatment with TOC fully prevents BAY-induced stimulation of autophagosome formation (Figure 3a). To further investigate the role of autophagosome formation in BAY-induced cell death, we performed knockdown of autophagy-related 5 (ATG5; Supplementary Figure S3B). This protein is required for autophagosome formation^26^ and its knockdown significantly reduced the BAY-induced increase in green puncta (Figure 3a). Remarkably, ATG5 knockdown also prevented the ROS increase after 24 h BAY treatment (Figure 3b). Analysis of cellular mitochondrial content using Mitotracker Green FM (MG) revealed that BAY treatment reduced MG staining and that this reduction was prevented by ATG5 knockdown (Figure 3c). This suggests that BAY-induced stimulation of ATG5-dependent autophagosome formation mediates mitochondria-specific autophagy (*i.e.* mitophagy). ATG5 knockdown inhibited BAY-induced loss of cell viability (Figure 3d). Taken together, these data suggest that TOC-sensitive 'triggering ROS' is required for mPTP opening and subsequent ATG5-mediated autophagosome formation. Moreover, our results suggest that ATG5-mediated autophagosome formation is required for sustained elevated ROS and increased mitophagy and eventually BAY-induced cell death.

### PINK1 knockdown inhibits BAY-stimulated mitophagy, Δ*ψ* depolarization, ROS increase and cell death

**Figure 4:**
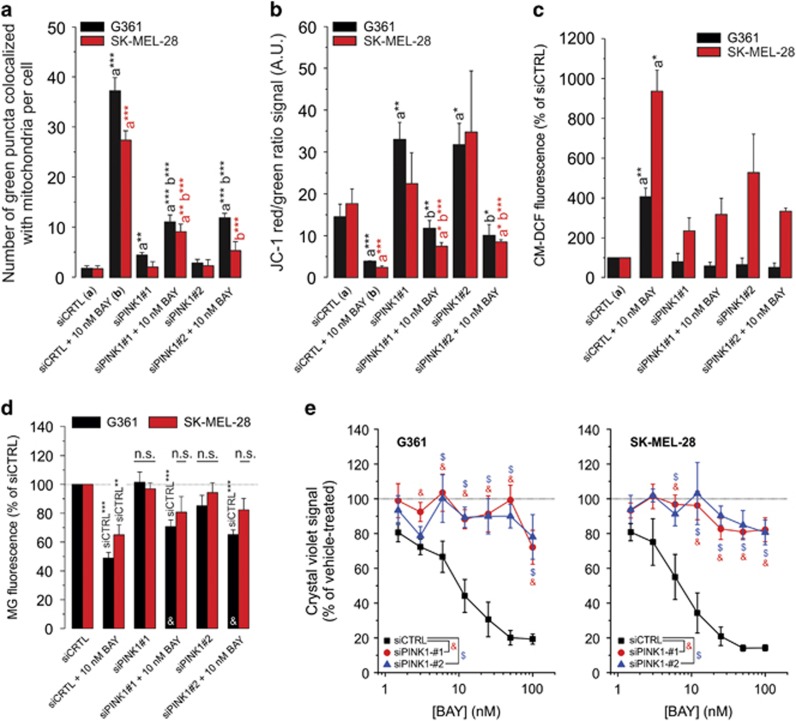
Effect of PINK1 knockdown on the BAY-induced stimulation of mitophagy, Δ*ψ* depolarization, reactive oxygen species (ROS) levels and reduction in cell viability. (**a**) Effect of BAY treatment, siPINK1-no. 1 and siPINK1-no. 2 on the number of green puncta colocalizing with mitochondria in G361 and SK-MEL-28 cells (at 24 h; *N*=3, *n*≥14). (**b**) Similar to panel a, but now for the effect on mitochondrial membrane potential (i.e. the JC-1 red/green ratio signal; *N*=3, *n*=6). (**c**) Similar to panel a, but now for the effect on cellular ROS levels (*N*=3, *n*=6). (**d**) Similar to panel a, but now for the effect on cellular MG fluorescence intensity (G361: at 24 h; SK-MEL-28: at 24 h; *N*=3, *n*=9). (**e**) Effect of BAY on the viability of cells (G361: at 48 h; SK-MEL-28: at 72 h; *N*=3, *n*=6) transfected with siCTRL, siPINK1-no. 1 and siPINK1-no. 2. Statistics: Significant differences relative to the indicated conditions are marked by *P<0.05, **P<0.01 and ***P<0.001. In panel c statistical analysis was performed using a one-sample t-test against a value of 100. In panel d, NS indicates nonsignificant and significant differences with the (siCTRL+10 nM BAY condition) are marked by ‘&'. In panel (**e**) significant differences (*P*<0.05) between conditions are marked by symbols (&, $)

To demonstrate the potential involvement of mitophagy in BAY-induced cell death, cells were transfected with GFP-LC3 (marking autophagosomes) and stained with MitoTracker Red (MR) to highlight mitochondria. Then, the number of green GFP puncta colocalizing with MR was determined to quantify the amount of mitophagy (Supplementary Figure S4C; arrowheads). BAY treatment (24 h) stimulated mitophagy (Figure 4a) and induced Δ*ψ* depolarization (Figure 4b). Phosphatase and tensin homolog-induced putative kinase 1 (PINK1) is a key regulator of mitophagy that recruits autophagy receptors to mitochondria upon Δ*ψ* depolarization.^27^ PINK1 knockdown (Supplementary Figure S3C) inhibited the BAY-induced changes (Figures 4a–d) as well as the BAY-induced reduction in cell viability (Figure 4e). The extent of mitophagy induction was positively correlated with the degree of Δ*ψ* depolarization (Supplementary Figure S1C), compatible with the key role of Δ*ψ* depolarization in mitophagy induction.^28^ These results suggest that BAY induces Δ*ψ* depolarization, thereby stimulating PINK1-dependent mitophagy and an ensuing increase in cellular ROS levels.

### Drp1 knockdown induces mitochondrial filamentation and inhibits BAY-stimulated cell death

**Figure 5:**
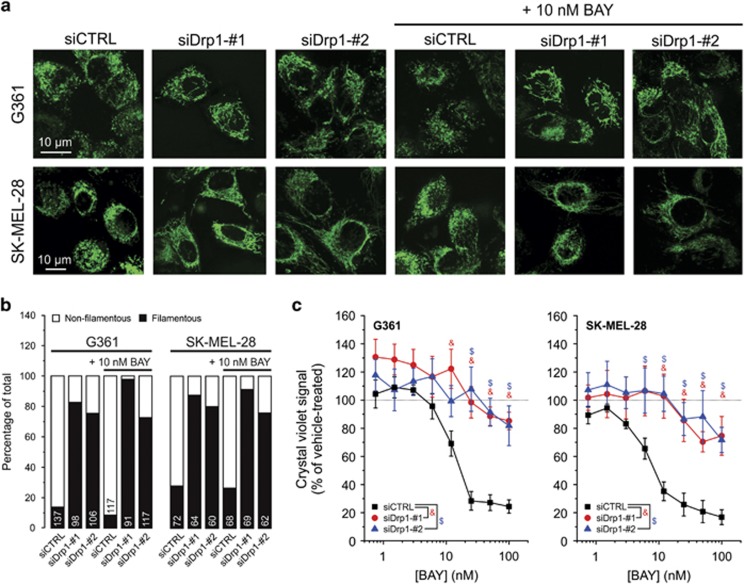
Effect of Drp1 knockdown on mitochondrial morphology and the BAY-induced reduction in cell viability. (**a**) Typical examples visualizing mitochondria morphology in MG-stained cells treated with siCTRL, siDrp1-no. 1 and siDrp1-no. 2 in the absence and presence of 10 nM BAY (G361: at 16 h; SK-MEL-28: at 24 h). (**b**) Quantification of mitochondrial morphology in multiple cells (*N*=2; numerals indicate the number of cells analyzed) for the conditions in panel (**a**). (**c**) Effect of BAY on the viability of cells (G361: at 48 h; SK-MEL-28: at 72 h; *N*=3, *n*=6) transfected with siCTRL, siDrp1-no. 1 and siDrp1-no. 2. Statistics: In panel (**c**), statistically significant differences (*P*<0.05) between conditions are marked by symbols (&, $)

In a starvation model,^29^ autophagosomal degradation of mitochondria was prevented by mitochondrial elongation (i.e. filamentation) mediated by downregulation of dynamin-related protein 1 (Drp1). This GTPase is one of the key mediators of mitochondrial fission.^30^ Two Drp1 isoforms, the brain (‘b') and ubiquitous (‘u') form, were detected in G361 and SK-MEL-28 cells. Knockdown of both isoforms (Supplementary Figure S3D) induced mitochondrial filamentation, both in the absence and presence of BAY (Figures 5a and b). Drp1 knockdown also inhibited the BAY-induced reduction in cell viability (Figure 5c). Compatible with these results (Supplementary Figure S1D), BAY-induced cell death was inhibited by a chemical inhibitor of Drp1 activity (Mdivi1; Cassidy-Stone *et al.*^31^). Mdivi1 also inhibited the BAY-induced stimulation of mitophagy (Supplementary Figure S1E). These results suggest that BAY-induced mitophagy stimulation and cell killing are inhibited by mitochondrial filamentation.

### BAY-induced cell death involves necroptosis and ferroptosis rather than apoptosis

**Figure 6:**
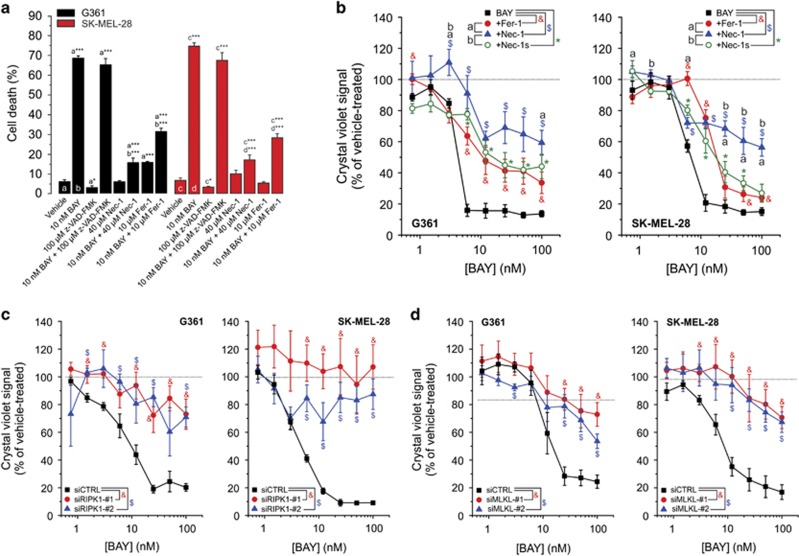
Effect of z-VAD-FMK, Nec-1, Nec-1s, Fer-1 and knockdown of RIPK1 and MLKL on the BAY-induced reduction in cell viability. (**a**) Effect of vehicle (*N*=5, *n*=15), BAY (*N*=5, *n*=15), the pancaspase inhibitor z-VAD-FMK (*N*=3, *n*=9), Nec-1 (*N*=3, *n*=9) and Fer-1 (*N*=3, *n*=9) on cell death (G361: at 48 h; SK-MEL-28: at 72 h). (**b**) Effect of BAY on viability (G361: at 48 h; SK-MEL-28: at 72 h; *N*=3, *n*=6) in the absence and presence of the ferroptosis inhibitor Fer-1 and the RIPK1 inhibitors Nec-1 and Nec-1s. (**c**) Effect of BAY on cell viability (G361: at 48 h; SK-MEL-28: at 72 h; *N*=3, *n*=6) in cells transfected with siCTRL, siRIPK1-no. 1 and siRIPK1-no. 2. (**d**) Effect of BAY on the viability of cells (G361: at 48 h; SK-MEL-28: at 72 h; N=3, *n*=6) transfected with siCTRL, siMLKl-no. 1 and siMLKL-no. 2. Statistics: Significant differences relative to the marked conditions are indicated by *P<0.05 and ***P<0.001. In panels b–d, significant differences (*P*<0.05) between conditions are marked by symbols (a, b, &, $)

**Figure 7:**
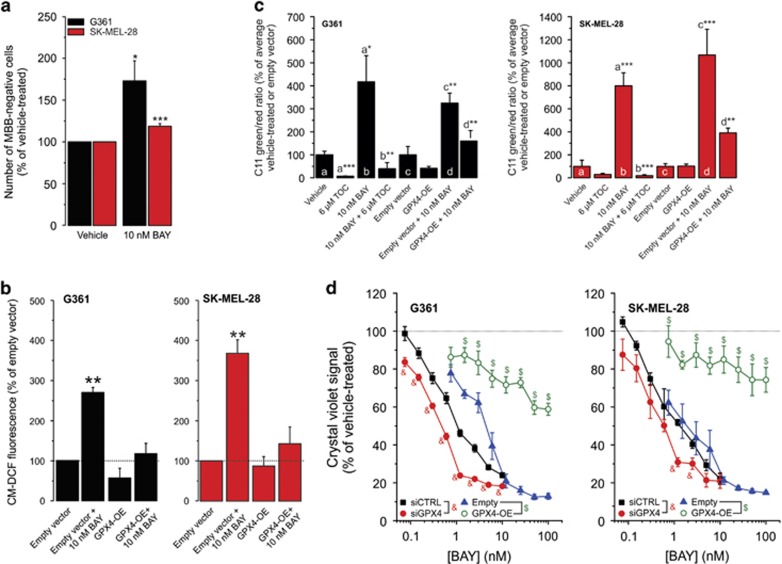
Effect of GPX4 knockdown/overexpression on the BAY-induced reduction in cell viability. (**a**) Effect of BAY on the number of MBB-negative (GSH-depleted) cells in the vehicle- and BAY-treated condition (G361: at 12 h; SK-MEL-28: at 24 h; *N*=3, *n*=9). Higher bars reflect a reduction in the number of MBB-positive cells. (**b**) Effect of BAY on reactive oxygen species (ROS) levels (at 24 h; *N*=3, *n*=3) in cells transfected with the empty or GPX4-OE vector. (**c**) Cellular lipid peroxidation (at 24 h) in vehicle-treated cells (G361: *N*=3, *n*=7; SK-MEL-28: *N*=3, *n*=6), TOC-treated cells (G361: *N*=3, *n*=7; SK-MEL-28: *N*=3, *n*=6), BAY-treated cells (G361: *N*=3, *n*=7; SK-MEL-28: *N*=3, *n*=6), BAY+TOC-treated cells (G361: *N*=3, *n*=7; SK-MEL-28: *N*=3, *n*=6), empty vector-transfected cells (G361: *N*=3, *n*=9; SK-MEL-28: *N*=3, *n*=8), GPX4-OE-transfected cells (G361: *N*=3, *n*=9; SK-MEL-28: *N*=3, *n*=8), empty vector-transfected-+BAY-treated cells (G361: *N*=3, *n*=9; SK-MEL-28: *N*=3, *n*=8) and GPX-OE transfected-+BAY-treated cells (G361: *N*=3, *n*=8; SK-MEL-28: *N*=3, *n*=8). (**d**) Effect of BAY on the viability of cells (G361: at 48 h; SK-MEL-28: at 72 h; *N*=3, *n*=6) transfected with siCTRL, siGPX4, empty vector and GPX4-OE. Statistics: Significant differences relative to vehicle (panel a), empty vector (panel b) and the marked conditions (panel c) are indicated by *P<0.05, **P<0.01, *P<0.01 and ***P<0.001. In panel d, significant differences (*P*<0.05) between conditions are marked by symbols (&, $)

Stimulation of autophagy was linked to induction of both apoptotic and necroptotic cell death.^32, 33, 34, 35, 36^ The broad-spectrum caspase inhibitor z-VAD-FMK was unable to prevent BAY-induced cell death (Figure 6a), arguing against involvement of apoptosis. On the other hand, Nec-1, which blocks necroptosis by inhibiting the activity of receptor-interacting serine/threonine-protein kinase 1 (RIPK1),^37, 38^ inhibited BAY-induced cell death (Figures 6a and b). However, Nec-1 not only inhibits RIPK1 but also indoleamine-2,3-dioxygenase, which catalyzes the conversion of tryptophan into kynurenine.^39, 40^ Therefore, we next investigated the effect of Nec-1s (or 7-Cl-O-Nec-1), a RIPK1 inhibitor displaying increased stability and specificity.^39, 41^ Nec-1s also inhibited BAY-induced cell death, albeit to a lesser extent than Nec-1 (Figure 6b), suggesting that this death is partially mediated by necroptosis. Compatible with this conclusion, knockdown of RIPK1 inhibited the BAY-induced loss in cell viability (Supplementary Figure S3E and Figure 6c). However, RIPK1 kinase activity is not only required for necroptosis but also for apoptosis.^42^ For this reason, we performed knockdown of the mixed lineage kinase domain-like protein (MLKL), the presence of which is essential for necroptosis induction.^41, 43^ Similar to RIPK1, MLKL knockdown inhibited the cell viability loss in BAY-treated cells (Supplementary Figure S3F and Figure 6d). Nec-1s only partially blocked BAY-induced cell death (Figure 6b), suggesting that necroptosis is not the only death mechanism involved. In this sense, autophagy can also promote ferroptosis,^44^ a mode of cell death that is negatively regulated by glutathione peroxidase 4 (GPX4) and characterized by increased iron-dependent ROS production, glutathione (GSH) depletion and lipid peroxidation.^41^ Supporting the involvement of ferroptosis, BAY treatment increased cytosolic ROS levels (see above), reduced cellular GSH levels (Figure 7a) and stimulated TOC-sensitive lipid peroxidation (Figure 7c). Moreover, we previously demonstrated that cotreatment with the GSH precursor *N*-acetyl cysteine inhibited the BAY-induced increase in cellular ROS levels and cell death.^19^ The ferroptosis inhibitor ferrostatin-1 (Fer-1; Dixon *et al.*^45^) partially prevented BAY-induced cell death (Figures 6a and b). Overexpression of the ferroptosis-inhibiting enzyme GPX4 (Supplementary Figure S3G) inhibited the BAY-stimulated increase in cellular ROS levels (Figure 7b) and lipid peroxidation (Figure 7c). In agreement with this result, GPX4 overexpression and knockdown (Supplementary Figure S3G) inhibited and potentiated the BAY-induced reduction in cell viability, respectively (Figure 7d). Overall, these results suggest that BAY treatment does not induce apoptotic cell death but triggers combined necroptosis and ferroptosis.

## Discussion

**Figure 8:**
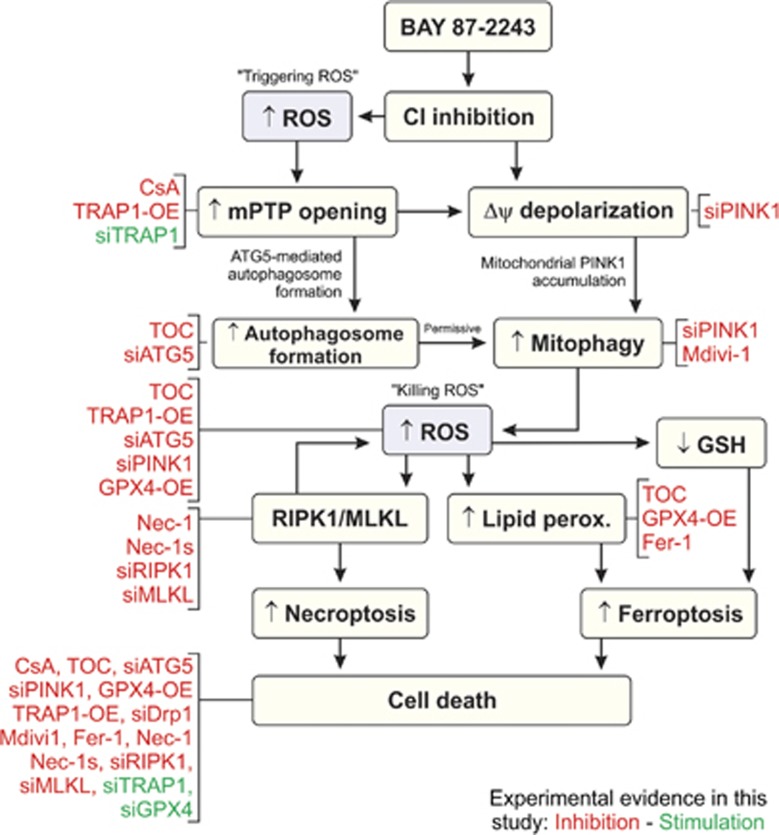
Proposed mechanistic model and experimental evidence. Treatment with BAY inhibits mitochondrial CI. This induces a (local) increase in reactive oxygen species (ROS) levels (‘triggering ROS'), which stimulates mPTP opening and autophagosome formation. Simultaneously, CI inhibition induces depolarization of the mitochondrial membrane potential (Δ*ψ*) leading to mitophagy induction. The latter increases ROS levels (‘killing ROS') leading to parallel stimulation of necrosome formation (RIPK1/MLKL), lipid peroxidation and GSH depletion. Increased necrosome formation further stimulates ROS levels and leads to induction of necroptosis, whereas lipid peroxidation and GSH depletion stimulate ferroptosis. Experimental evidence presented in this study are marked in red (inhibitory effect) and green (stimulatory effect)

Here we provide mechanistic insight into how BAY-induced inhibition of mitochondrial CI induces death in BRAF^V600E^ melanoma cells. A chain of events is proposed (Figure 8), in which CI inhibition stimulates mPTP opening, Δ*ψ* depolarization, autophagosome formation and mitophagy induction. The latter increases cellular ROS levels that stimulate lipid peroxidation and GSH depletion, leading to combined necroptotic and ferroptotic cell death.

### Specificity of BAY for melanoma cells

Within the used timeframe, BAY-induced CI inhibition effectively killed G361 and SK-MEL-28 melanoma cells, without affecting the viability of non-cancer cells. It was proposed that cancer cells displaying a higher basal ROS level than normal cells can be therapeutically targeted by ROS-inducing anticancer agents.^46^ This suggests that BAY treatment increases ROS levels beyond a death-inducing threshold in cancer cells but not in non-cancer cells. Compatible with this idea, we observed that G361 cells displayed a fourfold higher basal ROS level than SK-ML-28 cells (Supplementary Figure S1F). This might explain why BAY-induced killing occurred at a much earlier time point in G361 cells than in SK-MEL-28 cells.

### Role of external glucose

We previously demonstrated in C2C12 myoblasts that acute (30 min) inhibition of CI or CIII stimulates glycolytic ATP production to prevent ATP shortage.^47, 48^ Similarly, chronic (5 weeks) CI inhibition induced a fully glycolytic phenotype in primary skin fibroblasts, associated with an extreme sensitivity to glucose withdrawal.^49^ These findings led us to propose that BAY-induced CI inhibition might, in addition to increasing cellular ROS levels, induce a shortage of glucose contributing to cell death.^19^ Compatible with this idea and our current results, evidence was provided that chronic glucose depletion induces autophagic cell death in B16F1 melanoma cells.^50^ Moreover, we previously observed that increasing the external glucose concentration from 5 to 25 mM inhibited BAY-induced cell death.^19^ Here we demonstrate that regular medium refreshment does not markedly inhibit the BAY-induced reduction in cell viability, arguing against glucose shortage having a role in this process. In this sense, our observation that 25 mM glucose also reduces the BAY-induced ROS increase^19^ suggests that the inhibitory effect of high external glucose on BAY-induced cell death is ROS-mediated.

### Role of mPTP opening

Our results highlight an important role for mPTP opening in the death-inducing mechanism of BAY. TRAP1 overexpression also reduced the BAY-induced increase in cellular ROS levels. Supported by evidence in the literature,^22, 51^ this suggests that (part of the generated) ROS acts as an upstream activator of mPTP opening (Figure 8: ‘triggering ROS'). Inhibition of BAY-induced cell death by TOC might be due to this antioxidant preventing mPTP activation (by scavenging the ‘triggering ROS'). Compatible with this explanation, preliminary evidence suggests that the TOC derivative Trolox inhibits mPTP opening in primary human skin fibroblasts (Werner J.H. Koopman, personal observation). Alternatively, TOC might prevent cell death induction by lowering the levels of mitophagy-induced ‘killing ROS' (Figure 8). TRAP1 knockdown potentiated the BAY-induced reduction in cell viability, likely caused by increased ROS levels.^52^ This potentiation, in combination with the protective effect of TRAP1 overexpression, suggests that endogenous TRAP1 has a role in protecting the cells against BAY-induced cell death but that these levels and/or TRAP1 activity are insufficiently high. PINK1 can phosphorylate TRAP1 to prevent apoptosis induced by oxidative stress.^53^ This would mean that PINK1 knockdown should reduce TRAP1 activity, thereby potentiating BAY-induced cell death. In contrast, we observed that PINK1 knockdown antagonized the effects of BAY, arguing against PINK1 acting via TRAP1.

### Role of autophagosome formation and mitophagy

BAY treatment stimulated autophagosome formation, which was inhibited by the antioxidant TOC. In parallel, TOC also inhibited the BAY-induced increase in cellular ROS levels and lipid peroxidation. This suggests that increased ROS and/or lipid peroxidation are required for stimulation of autophagosome formation, supported by previous findings.^28, 54, 55, 56^ Upon Δ*ψ* depolarization, mitophagy is triggered by PINK1 accumulation on the surface of mitochondria ultimately leading to their autophagosomal uptake and lysosomal degradation.^57^ Knockdown of ATG5 and PINK1 inhibited autophagosome formation and mitophagy, respectively, suggesting that BAY-induced mPTP opening stimulates autophagosome formation,^24, 26^ allowing subsequent mitochondrial removal by mitophagy.^27^ Supporting this mechanism is the fact that mitochondrial content was reduced by up to 50% in BAY-treated cells and that this reduction was inhibited by knockdown of ATG5 or PINK1. Importantly, both ATG5 and PINK1 knockdown prevented the BAY-induced increase in cellular ROS levels and cell viability reduction. Stimulation and inhibition of autophagy in an angiogenesis model also increased and decreased ROS levels, respectively.^58^ This strongly suggests that increased cellular ROS levels and triggering of cell death are downstream effectors of autophagy/mitophagy (Figure 8). Although it might be possible that mitochondria produce increased amounts of ROS at some time during their mitophagic degradation, the exact mechanism by which autophagy/mitophagy increases ROS levels requires further investigation. PINK1 knockdown/knockout has been associated with mitochondrial fragmentation, increased ROS levels, Δ*ψ* depolarization and stimulation of mPTP opening.^59, 60, 61^ In contrast, here we observed that PINK1 knockdown by itself induced apparent Δ*ψ* hyperpolarization and, consistently, inhibited the BAY-induced stimulation of mitophagy, Δ*ψ* depolarization and ROS increase. These effects might be a melanoma- or context-specific phenomenon that, to the best of our knowledge, was not described previously. Whether mitophagy requires prior Drp1-mediated mitochondrial fragmentation is still controversial.^62^ For instance, mitochondrial division during mitophagy can occur in a Drp1-independent manner.^63^ On the other hand, Drp1-dependent mitochondrial fragmentation might facilitate mitophagy by creating small-size fragments facilitating autophagosomal uptake.^62^

### Role of mitochondrial morphology

We demonstrated that mitochondria display a non-filamentous morphology in G361 and SK-MEL-28 cells and that BAY-induced cell death is not associated with detectable changes in this morphological phenotype. Drp1 knockdown induced a filamentous mitochondrial morphology that was not affected by BAY treatment and also inhibited the BAY-induced reduction in cell viability. BAY-induced mitophagy and cell death were reduced by Mdivi1, a chemical inhibitor of Drp1 activity. These results suggest that mitochondrial filamentation prevents BAY-induced stimulation of mitophagy and thereby cell death. In parallel, mitochondrial filamentation might also mitigate mitochondrial dysfunction by damage and/or antioxidant sharing,^64^ thereby inhibiting the ROS trigger for mPTP-induced mitophagy induction (Figure 8). In addition to its role in mitophagy PINK1 is a known regulator of the mitochondrial fission and fusion machinery. Using primary neurons and COS-7 cells, it was demonstrated that PINK1 knockdown induces mitochondrial filamentation.^65^ This might suggest that in our study part of the inhibitory effect of PINK1 knockdown on BAY-induced cell death is mediated by stimulation of mitochondrial filamentation. Interestingly, melanoma cells that were made resistant against the BRAF^V600E^-specific inhibitor and chemotherapy drug Vemurafenib possessed filamentous mitochondria.^66^ Conversely, knockdown of the fusion-promoting protein mitofusin 2 in melanoma cells induced a more fragmented mitochondrial phenotype and increased cell death upon Vemurafenib treatment.^67^ Therefore, our results might suggest that prevention or reversal of mitochondrial filamentation could be a strategy to overcome Vemurafenib resistance in BRAF^V600E^ melanoma cells.

### Mode of cell death

We observed that z-VAD-FMK neither displayed cytotoxic effects by itself nor prevented BAY-induced cell death, arguing against involvement of apoptosis. The inhibitory effects of Nec-1s and Fer-1 suggest that combined necroptosis and ferroptosis are responsible for the BAY-induced reduction in cell viability. Involvement of necroptosis is further supported by the inhibitory effects of RIPK1 and MLKL knockdown on the BAY-induced reduction in cell viability. Compatible with the proposed mechanism (Figure 8), increased ROS levels have been demonstrated to promote stabilization of the RIPK1/RIPK3 necrosome.^68^ The latter study also provided evidence that RIPK1, RIPK3 and MLKL stimulate ROS production, which further promotes necrosome stabilization. Involvement of ferroptosis is supported by the observation that BAY treatment increases ROS levels, stimulates lipid peroxidation and induces GSH depletion, all of which are hallmarks of ferroptotic cell death.^41^ It was recently demonstrated that oxidation of specific phosphatidylethanolamines (PEs) in endoplasmic-reticulum-associated cell compartments depends on acyl-CoA synthase 4 and is required for ferroptosis induction.^69, 70^ Compatible with our TOC results, tocopherols suppressed ferroptosis by inhibiting lipoxygenase, which generates doubly and triply oxygenated (15-hydroperoxy)-diacylated PE species that act as death signals.^69, 70^ Knockdown and overexpression of GPX4, an essential regulator of ferroptotic cell death,^69, 70, 71^ potentiated and antagonized the BAY-induced reduction in cell viability, respectively. This again suggests involvement of ferroptosis in BAY-induced cell death. It is currently unclear how increased cellular ROS levels induce specific activation of necroptosis or ferroptosis.^72^ Nec-1s and Fer-1 inhibited the BAY-induced reduction in cell viability to a similar extent, suggesting that both death mechanisms are equally activated. Erastin-induced ferroptosis was promoted by autophagic degradation of ferritin and ATG5 knockdown inhibited this ferroptosis.^44^ Therefore, BAY-induced stimulation of autophagy might directly activate ferroptosis. However, knockdown of ATG5 or PINK1 prevented the BAY-induced reduction in cell viability to a similar extent, suggesting that PINK1-dependent mitophagy, occurring downstream of ATG5-dependent autophagosome formation, is primarily responsible for cell death induction.

## Conclusion

Using BAY as a tool, we here provide evidence that CI inhibition induces the death of BRAF^V600E^ melanoma cells by stimulating mPTP opening and inducing Δ*ψ* depolarization. These events increase cellular ROS levels in a mitophagy-dependent manner, leading to induction of combined necroptotic/ferroptotic cell death.

## Materials and methods

### Cell culture

BRAF^V600E^ human melanoma cell lines G361 (no. ATCC-CRL-1424) and SK-MEL-28 (no. ATCC-HTB-72) were derived from a skin melanoma site and obtained from American Type Culture Collection (ATCC; LGC Standards GmbH, Wesel, Germany). Melanoma cells were routinely cultured at 37 °C and 5% CO_2_ in ATCC-recommended media supplemented with 10% (v/v) fetal calf serum (FCS; Gibco-Thermo-Fisher, Waltham, MA, USA). Before experiments, the above cells were cultured for 12 h in a pyruvate-free medium (no. A1443001; Gibco Thermo Fisher Scientific Inc., Waltham, MA, USA) to which was added: 5 mM d-glucose (Sigma-Aldrich, St. Louis, MO, USA), 2 mM GlutaMAX (no. 35050038; Gibco) and 5% (v/v) dialyzed FCS (no. 26400044; 10 000 MW cutoff; Gibco). Cells were routinely cultured in a humidified atmosphere (95% air, 5% CO_2_, 37 °C).

### Knockdown/overexpression studies and western blot analysis

Knockdown/overexpression was performed as described in the Supplementary Information. For western blotting, cell pellets were lysed in modified RIPA buffer (150 mM sodium chloride, 1% Triton X-100, 0.5% sodium deoxycholate, 0.1% SDS, 50 mM Tris, pH 8.0) containing 1x protease inhibitor cocktail (no. 116974898001; Roche, Mannheim, Germany). Protein concentration in the lysates was determined using a NanoDrop UV-Vis Spectrophotometer (Thermo Fisher Scientific Inc., Wilmington, DE, USA), and 100 *μ*g of protein per well was separated by SDS-PAGE and transferred to a PDVF membrane using an iBlot gel transfer stack (Invitrogen, Carlsbad, CA, USA). Antibodies and their detection are described in the Supplementary Information.

### Cell viability, death assay and mPTP opening

Cell viability was determined using crystal violet staining. Cell death was assessed by flowcytometric analysis of propidium iodide (PI)-stained cells using a FACSCalibur flow cytometer (BD Biosciences, Breda, The Netherlands). Analysis of mPTP opening was performed using a previously described approach.^22^ Additional information is provided in the Supplementary Information.

### Quantification of autophagy, mitophagy and mitochondrial morphology

For autophagy analysis, cells were seeded on Nunc Lab-Tek glass-bottomed coverslips (no. 565470; Thermo Scientific), transfected with a construct encoding tandem mCherry-GFP-tagged LC3 (no. 21074; Addgene, Cambridge, MA, USA) and cultured for 24 h. Mitophagy was analyzed by transfecting the cells with a GFP-LC3 construct (no. P36235; Invitrogen), followed by subsequent costaining with MR (Invitrogen). Mitochondrial morphology was analyzed by staining the cells with MG (Invitrogen). Details are provided in the Supplementary Information.

### Analyses of cellular ROS levels, GSH levels, lipid peroxidation, mitochondrial membrane potential and mitochondrial content

To measure ROS and GSH levels, cells were stained with CM-H_2_DCFDA or monobromobimane (MBB), respectively. Cellular lipid peroxidation was quantified using C11-BODIPY (‘C11'). Mitochondrial membrane potential was quantified using 5,5′,6,6′-tetrachloro-1,1′,3,3′-tetraethylbenzimidazolyl-carbocyanine iodide (‘JC-1') staining. Mitochondrial content was determined using MG staining. Details are provided in the Supplementary Information.

### Chemicals

*N*-benzyloxycarbonyl-Val-Ala-Asp-fluoromethylketone (no. ALX260020; Z-VAD-FMK) was obtained from Enzo Life Sciences (Raamsdonkveer, The Netherlands) and Nec-1 (no. SC200142) from Santa Cruz Biotech (Dallas, TX, USA). (+)-TOC (no. T3251), BafA1 (no. B1793), Crystal Violet solution (no. HT90132), CsA (no. 30024), Fer-1 (no. SML0583), Mdivi1 (no. M0199) and PI (no. P4170; PI) were obtained from from Sigma-Aldrich. Nec-1s (no. HY-14622A) was purchased from Bio-Connect BV (Huissen, The Netherlands). BAY) was provided by Bayer AG (Leverkusen, Germany).

### Data and image analysis

The number of independent experiments (days) and replicates (assays, individual cells) are marked by *N* and *n*, respectively. Unless stated otherwise, statistical significance was assessed using an independent two-population Student's *t*-test (*P<0.05; **P<0.01; ***P<0.001; relative to the indicated condition), and results from multiple experiments are represented by their average value±S.E.M. Curve fitting and statistical analysis was performed using Origin Pro 6.1 (OriginLab, Northampton, MA, USA). Image visualization, processing and quantification was carried out using Image Pro Plus 6.1 (Media Cybernetics, Rockville, MD, USA) and FIJI software (HTTP://fiji.sc/).
