# Supplementary material for: Mitochondrial complex I inhibition triggers a mitophagy-dependent ROS increase leading to necroptosis and ferroptosis in melanoma cells
Source: Cell Death Dis. 2017 Mar 30;8(3):e2716–. doi: 10.1038/cddis.2017.133 (PMC5386536; doi:10.1038/cddis.2017.133)
Supplement: Supplementary Information [file cddis2017133x1.pdf]

A. SUPPLEMENTARY MATERIALS AND METHODS

Additional cell lines - Human epidermal melanocytes from lightly pigmented adult skin (Hema-LP, #C-024-5C) were obtained from Gibco (**Gibco-Thermo-Fisher, Waltham, MA, USA**) and cultured in Medium 254 (#M254500; **Gibco**). This medium was supplemented with Human Melanocyte Growth Supplement-2 (HMGS-2; #S0165; **Gibco**). Human primary skin fibroblasts (CT5120) were obtained from a healthy individual according to the relevant Institutional Review Boards and cultured in HEPES-buffered (25 mM) medium 199 (M199) with Earle's salt supplemented with 10% (v/v) FCS (**Gibco**), 100 IU/ml penicillin and 100 IU/ml streptomycin (**Invitrogen, Breda, The Netherlands**). M199 medium also contained 5.5 mM glucose, 0.7 mM glutamine and lacked pyruvate. Cells were routinely cultured in a humidified atmosphere (95% air, 5% CO_2, 37° C).

Knockdown and overexpression studies - For RNA interference, cells were transfected with SilencerSelect siRNA (**Invitrogen**), control siRNA (4390843), siRNA targeting RIPK1 (siRIPK1#1: s16651; siRIPK1#2: s16653), siRNA targeting PINK1 (siPINK1#1: s35166 and siPINK1#2: s35168), siRNA targeting ATG5 (siATG5#1: s18158 and siATG5#2: 18160), siRNA targeting Drp1 (siDrp1#1: s19559 and siDrp1#2: s19560), siRNA targeting MLKL (siMLKL#1: s47087: and siMLKL#2: s47088), siRNA targeting GPX4 (s6110) or siRNA targeting TRAP1 (s177). SiRNA was transfected into the cell using the Neon transfection system (**Invitrogen**) according to the manufacturer's instructions. Transient overexpression of the murine GPX4-pcDNA3 construct and stable overexpression of pCMV6-AC-TRAP1 (**Origene, Uden, The Netherlands**) was also performed using the Neon transfection system (**Invitrogen**). Electroporation was carried out with an electroporator (Neon with pipette station; **Invitrogen**) using 3 pulses (10 ms pulse width; 1350 V). Cells were subsequently selected by culturing them in the presence (200 µg/ml) of the selective antibiotic Geneticin® (G418) sulfate (#11811023; **Gibco**) during 1 wk.

Western blot antibodies and detection - The PVDF membrane was probed with mouse anti-GPX4 (#MAB5457; **R&D Systems Inc., Minneapolis, MN, USA**), rabbit anti-TRAP1 (#HPA044227; **Sigma-Aldrich**), rabbit anti-Drp1 (#ABT155; **Sigma-Aldrich**), rabbit anti-MLKL (#SAB2108746; **Sigma-Aldrich**), rabbit anti-ATG5 (#2630; **Cell Signaling Technology, Danvers, MA, USA**), rabbit anti-PINK1 (#6946P; **Cell Signaling**), mouse anti-RIPK1 (#610458; **BD Biosciences, San Jose, CA, USA**), and mouse β-actin (#A5541; **Sigma-Aldrich**), followed by goat-anti-mouse or goat-anti-rabbit labeled with IRDye infrared dyes (**LI-COR Biosciences, Leusden, The Netherlands**). A LI-COR Odyssey CLX infrared imaging system (**LI-COR**) was used for fluorescence detection.

Cell viability assay - Cells were seeded in 24-well plates and subjected to the various treatments. Next, the cells were washed with PBS and incubated for 10 min at RT in a staining solution consisting of 0.5% (v/v) crystal violet solution, 30% (v/v) ethanol and 3% (v/v) formaldehyde. After staining, plates are rinsed with water followed by addition of a 1% (w/v) SDS solution for 30 min on an orbital shaker. The amount of re-solubilized crystal violet can be used as a readout of relative cell numbers (**Śliwka et al., 2016**) by absorbance measurements (550 nm) using a microplate spectrophotometer (**Benchmark Plus, Biorad, Veenendaal, The Netherlands**).

Cell death assay - Cell death was assessed by flowcytometric analysis of propidium iodide (PI)-stained cells using a FACSCalibur flow cytometer (**BD Biosciences**) as described previously (**Riccardi & Nicoletti, 2006**). In the PI staining histogram, sub-G1 cells displayed a broad hypodiploid peak that can

be easily discriminated from the narrow peak representing cells with diploid (normal) DNA content (**Fig. S1G**). The sub-G1 cell population is suited to quantify apoptotic and non-apoptotic forms of cell death (**Dächert et al., 2016; Gerges et al., 2016; Hannes et al., 2016; Riccardi & Nicoletti, 2006**).

Live-cell microscopy analysis of photoinduced mPTP opening - Cells were seeded onto Ø24 mm-diameter glass coverslips (#6311345; **VWR International B.V., Amsterdam, NL**) and cultured for 12 h. Next, cells were incubated with 15 nM Tetramethylrhodamine methylester (TMRM, **Invitrogen**) in collected culture medium (25 min, 37° C, 5% CO_2 in the dark). After incubation, the cells were washed three times with PBS and placed in a colorless HEPES-Tris (HT) buffer (containing 132 mM NaCl, 10 mM HEPES, 4.2 mM KCl, 1 mM $MgCl_2$, 1 mM $CaCl_2$ and 5 mM D-glucose, adjusted to pH=7.4 with Tris salt). The coverslips were firmly mounted on the temperature-controlled (37 °C) stage of an inverted microscope (**Axiovert 200M, Carl Zeiss, Jena, Germany**) equipped with a Zeiss x40 1.3 NA Fluar objective. TMRM was excited at 540 nm using a monochromator (**Polychrome IV, TILL Photonics, Gräfelfing, Germany**) and emission signals were directed to a CoolSNAP HQ monochrome CCD-camera (**Roper Scientific, Vianen, The Netherlands**) using a 560DRLP dichroic mirror (XF2017; **Omega Optical Inc., Brattleboro, VT, USA**) and 565ALP emission filter (XF3085; **Omega**). Microscopy hardware was controlled using Metafluor 6.0 software (**Universal Imaging Corporation, Downingtown, PA, USA**). Next, images were recorded during 15 min using an interval of 1 s and a 150 ms excitation time per image. This imaging regime induces reversible mPTP openings that are photoinduced by controlled illumination of TMRM. Applying our published protocol, images 400-450 were used to count the number of reversible mPTP openings (**Blanchet et al., 2014**). The analysis process is illustrated in **Supplementary Fig. S2**, the images of which are derived from the **Supplemental movie**. This typical experiment depicts TMRM-stained G361 cells transfected with an empty vector (**Supplementary Fig. S2A**; left column), the difference images (calculated by subtracting the $(n-1)^{th}$ image from the nth image in the recorded TMRM image sequence; middle column) and a colorized version of the difference image to visualize the fluorescence changes (right column). For quantification, individual mPTP openings were manually counted from the difference image (dark pixels marked by arrowheads in **Supplementary Fig. S2A**), and visually cross-checked against changes in TMRM intensity (*i.e.* true mitochondrial depolarization linked to loss of TMRM staining). For each movie, the total number of counted mPTP opening events was normalized to the number of cells present in the field of view. In the provided example (**Supplementary Fig. S2A**), the cells required manual refocusing during recording. This induced upward and downward changes in the average intensity of the difference image (**Supplementary Fig. S2B**; marked 'R' and figure inset). Images with refocusing-induced artefacts were omitted from the analysis. At the end of the recording (image 902), the mitochondrial membrane potential was depolarized, as illustrated by the non-mitochondrial localization of TMRM (**Supplementary Fig. S2C**).

Quantification of autophagy and mitophagy using confocal microscopy - For autophagy analysis, cells were seeded on Nunc® Lab-Tek® glass-bottomed coverslips (#565470; **Thermo Scientific**), transfected with a construct encoding tandem mCherry-GFP-tagged LC3 (#21074; **Addgene, Cambridge, MA, USA**) and cultured for 24 h (**Kimura et al., 2007; Pankiv et al., 2007**). Mitophagy was analyzed by transfecting the cells with a GFP-LC3 construct (#P36235; **Invitrogen**), followed by subsequent co-staining with MitoTracker Red (MR; **Invitrogen**), as described previously (**Dagda et al., 2009**). Cells were fixed using a 4% (v/v) paraformaldehyde solution for 15 min at RT and visualized using confocal microscopy (SP-5; **Leica Microsystems GmbH, Wetzlar, Germany**). Images were acquired using a x63 water immersion objective with an NA of 1.2 (HCX-PL-APO). In cells transfected

with mCherry-GFP-tagged LC3, GFP and mCherry were excited at 488 nm and 561 nm, respectively, and emission was detected between 500-550 nm (GFP) and 600-680 nm (mCherry). In cells transfected with GFP-LC3 and stained with MR, GFP and MR were excited at 488 nm and 561 nm, respectively, and emission was detected between 500-550 nm (GFP) and 587-657 nm (MR). Image acquisition was controlled using Leica software (LAS AF Lite, version 2.6.3). The number of green puncta per cell (autophagy) and the number of green puncta per cell colocalized with mitochondria (mitophagy) were manually quantified for each image in a blinded fashion.

Analysis of cellular ROS levels and lipid peroxidation - To measure ROS levels cells were incubated with CM-H_2DCFDA (5 µM; **Invitrogen**) for 30 min at 37°C (**Koopman et al., 2006**). To analyse cellular lipid peroxidation, cells were incubated with C11-BODIPY ("C11"; 5 µM; **Invitrogen**) for 30 min at 37°C (**Distelmaier et al., 2015**). Directly after staining, the fluorescence signals of CM-H_2DCFDA- and C11-BODIPY-stained cells were analyzed using a FACSCalibur flow cytometer (**BD Biosciences**).

Analysis of cellular GSH levels - To determine GSH levels, cells were incubated with 40 µM monobromobimane (MBB; **Sigma**) for 30 min at 37° C and immediately analyzed using a FACSVerse flow cytometer (**Becton Dickinson (BD) Biosciences, East Rutherford, NJ, USA**). MBB is a thiol-reactive probe that is essentially non-fluorescent and alkylates thiol groups (**Winter & Thorpe, 2014**). This displaces the bromine and adds the fluorescent tag ($\lambda_{emission}$ = 478 nm) to the thiol group. Fluorescence data was analyzed on the live cell population using the FlowJo X software.

Analysis of mitochondrial membrane potential - Mitochondrial membrane potential was quantified using 5,5',6,6'-tetrachloro-1,1',3,3'-tetraethylbenzimidazolyl-carbocyanine iodide ("JC-1"). Mitochondrial accumulation of JC-1 (which displays a green fluorescence) is membrane potential dependent and leads to the formation of red fluorescent J-aggregates. The ratio between red/green emissions can be used as a measure of mitochondrial membrane potential (**Smiley et al., Proc. Natl. Acad. Sci. USA, 1991**). Cells were stained with JC-1 (1 µM; **Invitrogen**) for 30 min at 37°C and subsequently analyzed using FACSCalibur flow cytometer (**BD Biosciences**).

Analysis of cellular mitochondrial content - To analyze mitochondrial content, cells were stained with Mitotracker Green FM (MG; 100 nM; **Invitrogen**) for 30 min at 37°C and subsequently analyzed using a FACSCalibur flow cytometer (**BD Biosciences**). The MG fluorescence intensity was used as a measure of cellular mitochondrial content (**Schöckel et al., 2015; Haq et al., 2013**).

Analysis of mitochondrial morphology - Cells were seeded on Nunc® Lab-Tek® glass-bottomed coverslips (#565470; **Thermo Scientific**) and cultured for 24 h. Mitochondria were stained by incubating the cells in fresh culture medium without phenol red containing MitoTracker Green FM (MG; 100 nM, **Invitrogen**) for 45 min (37° C, 5% CO_2 in the dark). After incubation, cells were placed in fresh culture medium without phenol red, and visualized by confocal microscopy (SP-5; **Leica Microsystems GmbH, Wetzlar, Germany**). Images were acquired using a x63 water immersion objective with an NA of 1.2 (HCX-PL-APO). MG was excited by an argon laser (488 nm) and emission was collected between 500 and 550 nm. Recorded images were visually analyzed in a blinded fashion by scoring the number of cells displaying a filamentous or non-filamentous mitochondrial morphology.

B. SUPPLEMENTARY FIGURES

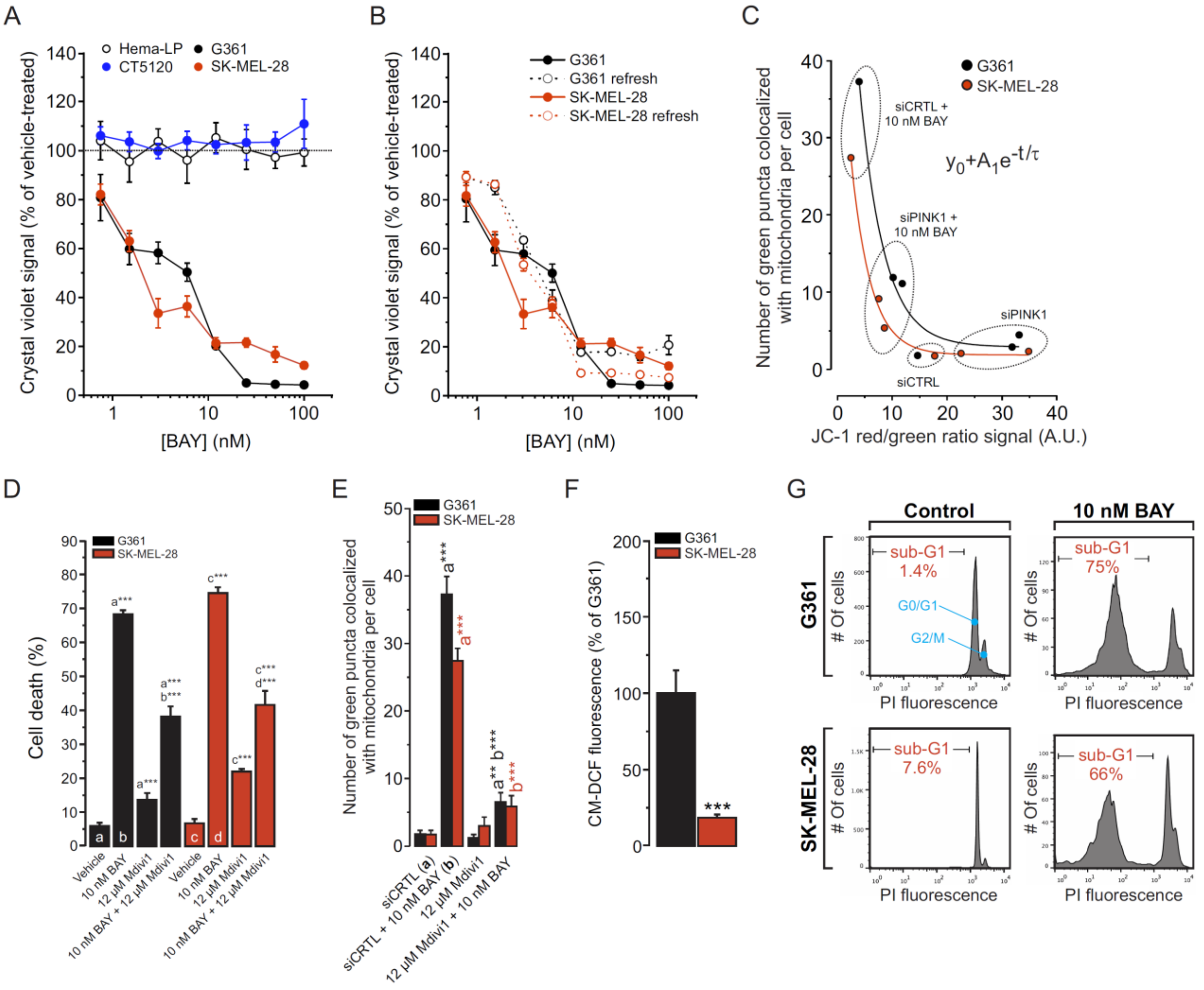

Supplementary Figure S1: (**A**) Dose-dependent effect of BAY (@72h; N=2, n=4) on the viability of Hema-LP cells, primary human skin fibroblasts (CT5120), G361 cells and SK-MEL-28 cells. (**B**) Dose-dependent effect of BAY (@72h; N=2, n=4) on the viability of G361 and SK-MEL-28 cells. The dotted lines and open symbols represent cells for which the extracellular medium was refreshed @24h and @48h. (**C**) Relationship between mitochondrial membrane potential (x-axis) and the amount of mitophagy (y-axis). A monoexponential relationship was used to fit the data (G361: R^2=0.97, τ=4.41±1.08; SK-MEL-28: R^2=0.99, τ=3.56±0.388). (**D**) Effect of vehicle, (N=5, n=15), BAY (N=5, n=15), the Drp1-inhibitor Mdivi1 (N=3, n=9) and Mdivi1+BAY (N=3, n=9) on cell death (G361: @48h; SK-MEL-28: @72h). (**E**) Effect of BAY and Mdivi1 on the amount of mitophagy in G361 and SK-MEL-28 cells (24 h treatment with 10 nM BAY; N=3, n≥14). (**F**) ROS levels in G361 and SK-MEL-28 cells (N=2, n=4). (**G**) Typical example of how the percentage of sub-G1 cells was quantified and used as a measure of cell death in PI-stained G361 and SK-MEL-28 cells in the absence (Control) and presence of 10 nM BAY (G361: @48h; SK-MEL-28: @72h). **Statistics:** Significant differences relative to the indicated conditions (a,b,c,d; Panel D-E) and G361 cell line (panel F) are indicated by **P<0.01 and ***P<0.001.

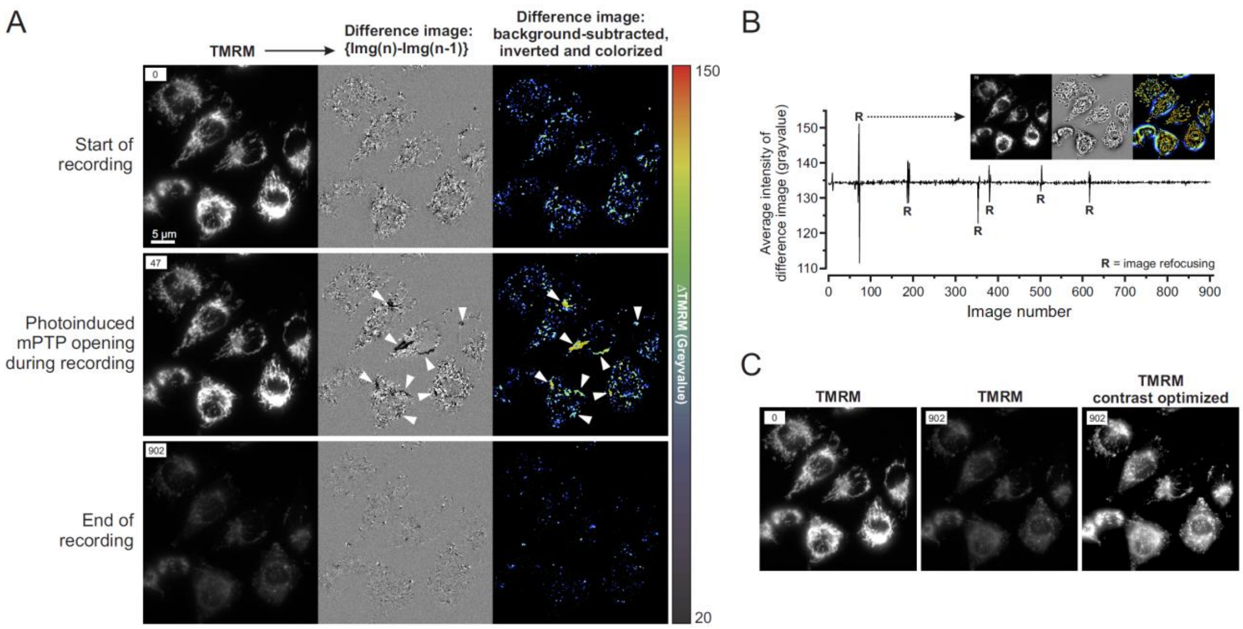

Supplementary Figure S2: Analysis protocol to quantify photo-induced mPTP openings. (**A**) This typical experiment (data taken from the **Supplemental movie**) depicts TMRM-stained G361 cells transfected with an empty vector (left column), the difference images (calculated by subtracting the (n-1)th image from the nth image in the recorded TMRM image sequence; middle column) and a colorized version of the difference image to visualize the fluorescence changes (right column and color scale bar on the right). To allow color-coding of the difference image it was first background-corrected followed by inverting the pixel values (*i.e.* black pixels become white and white pixels become black). The position of the image in the recorded sequence is marked at the top left of the TMRM image. (**B**) During the experiment, manual refocusing was required. This induced upward and downward changes in the average intensity of the difference image (marked 'R' and figure inset). (**C**) At the end of the recording (image 902), the mitochondrial membrane potential was depolarized, as illustrated by the non-mitochondrial localization of TMRM.

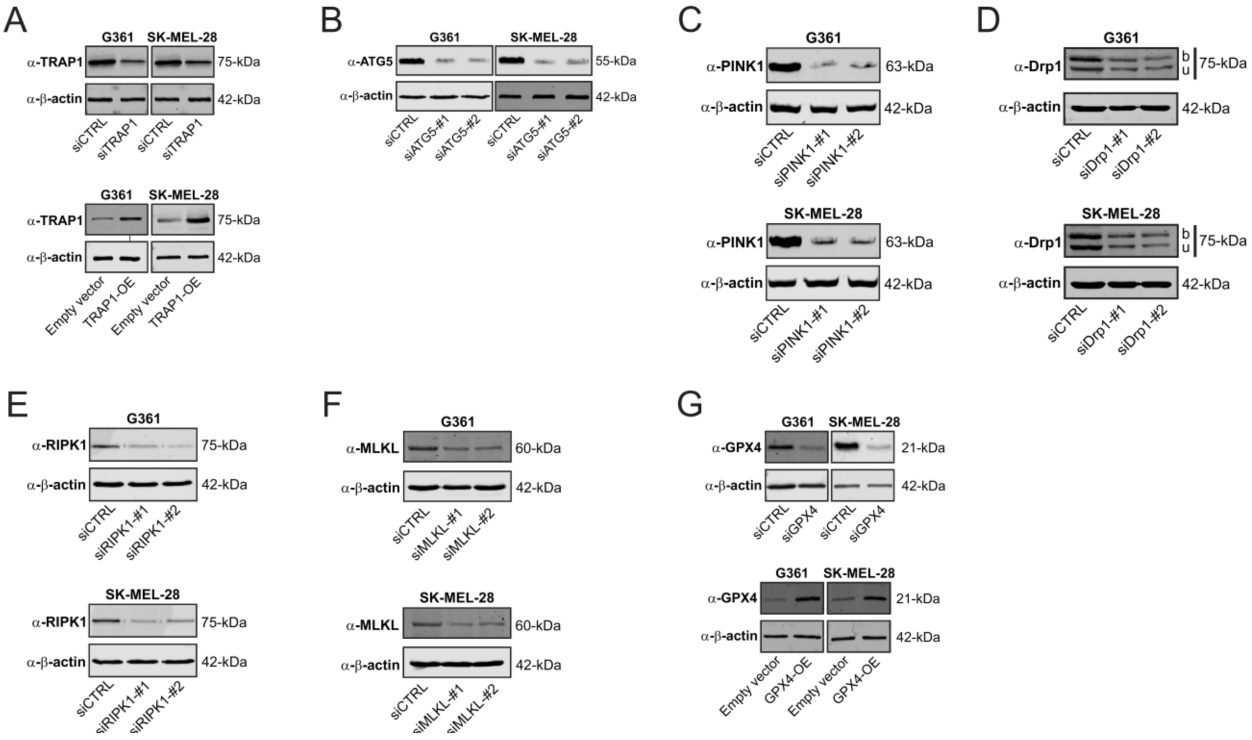

Supplementary Figure S3: Western blot analysis. (**A**) Western blot analysis depicting TRAP1 levels in cells transfected with a control siRNA (siCTRL), a TRAP-targeting siRNA (siTRAP1), an empty vector, and a TRAP1 overexpression construct (TRAP1-OE). β-actin was used as a loading control (G361: @48h; SK-MEL-28:@72h). (**B**) Same as panel A, but now for ATG5 levels in cells transfected with a control siRNA (siCTRL) and two ATG5-targeting siRNAs (siATG5-#1, siATG5-#2). (**C**) Same as panel A, but now for PINK1 levels in cells transfected with a control siRNA (siCTRL) and two PINK1-targeting siRNAs (siPINK1-#1, siPINK1-#2). (**D**) Same as panel A, but now for Drp1 levels in cells transfected with a control siRNA (siCTRL) and two Drp1-targeting siRNAs (siDrp1-#1, siDrp1-#2). Two Drp1 isoforms, the brain ("b") and ubiquitous ("u") form, were detected. (**E**) Same as panel A, but now for RIPK1 levels in cells transfected with a control siRNA (siCTRL) and two RIPK1-targeting siRNAs (siRIPK1-#1, siRIPK1-#2). (**F**) Same as panel A, but now for MLKL levels in cells transfected with a control siRNA (siCTRL) and two MLKL-targeting siRNAs (siMLKL-#1, siMLKL-#2). (**G**) Same as panel A, but now for GPX4 levels in cells transfected with a control siRNA (siCTRL), a GPX4-targeting siRNA (siGPX4), an empty vector, and a GPX4 overexpression construct (GPX4-OE).

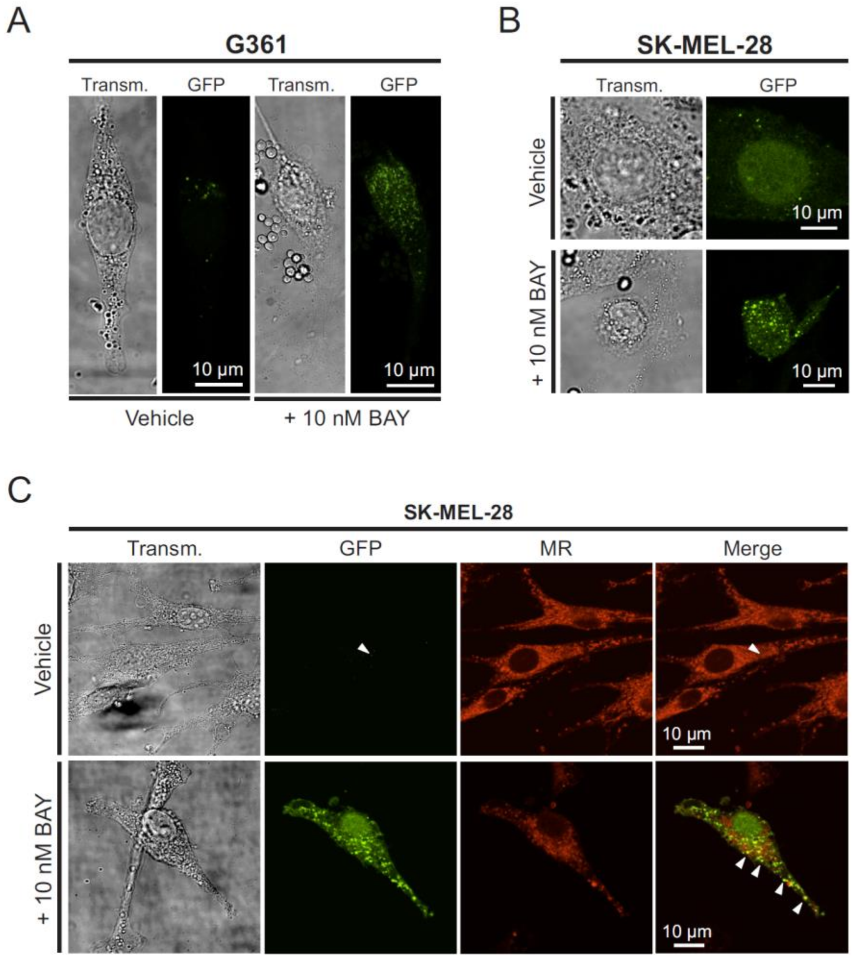

Supplementary Figure S4: Autophagy and mitophagy analysis. (**A**) Typical example showing autophagy stimulation (*i.e.* an increase in green GFP puncta) in G361 cells by BAY treatment (@24h). Also a transmission microscopy image (Transm.) is shown. (**B**) Similar to panel A, but now for SK-MEL-28 cells. (**C**) Typical example showing mitophagy stimulation (*i.e.* an increase in green GFP puncta colocalizing with the mitochondrial marker mitotracker Red) in SK-MEL-28 cells by BAY treatment (@24h). Also a transmission microscopy image (Transm.) is shown.

C. SUPPLEMENTARY REFERENCES

1. **Blanchet L**, Grefte S, Smeitink JA, Willems PH, Koopman WJ. Photo-induction and automated quantification of reversible mitochondrial permeability transition pore opening in primary mouse myotubes. *PloS one* 2014, **9**:e114090.
2. **Dächert J**, Schoeneberger H, Rohde K, Fulda S. RSL3 and Erastin differentially regulate redox signaling to promote Smac mimetic-induced cell death. *Oncotarget* 2016, **7**:63779-63792.
3. **Dagda RK**, Cherra SJ, 3rd, Kulich SM, Tandon A, Park D, Chu CT. Loss of PINK1 function promotes mitophagy through effects on oxidative stress and mitochondrial fission. *J. Biol. Chem.* 2009, **284**:13843-13855.
4. **Distelmaier F**, Valsecchi F, Liemburg-Apers DC, Lebiedzinska M, Rodenburg RJ, Heil S, et al. Mitochondrial dysfunction in primary human fibroblasts triggers an adaptive cell survival program that requires AMPK-alpha. *Biochim. Biophys. Acta.* 2015, **1852**:529-540.
5. **Gerges S**, Rohde K, Fulda S. Cotreatment with Smac mimetics and demethylating agents induces both apoptotic and necroptotic cell death pathways in acute lymphoblastic leukemia cells. *Cancer Lett.* 2016, **375**:127-132.
6. **Hannes S**, Abhari BA, Fulda S. Smac mimetic triggers necroptosis in pancreatic carcinoma cells when caspase activation is blocked. *Cancer Lett.* 2016, **380**:31-38.
7. **Haq R**, Shoag J, Andreu-Perez P, Yokoyama S, Edelman H, Rowe GC, et al., Oncogenic BRAF regulates oxidative metabolism via PGC1α and MITF. *Cancer Cell* 2013, 23(3): 302-315.
8. **Kimura S**, Noda T, Yoshimori T. Dissection of the autophagosome maturation process by a novel reporter protein, tandem fluorescent-tagged LC3. *Autophagy* 2007, **3**: 452-460.
9. **Koopman WJH**, Verkaart S, van Emst-de Vries SE, Grefte S, Smeitink JAM, Willems PHGM. Simultaneous quantification of oxidative stress and cell spreading using 5-(and-6)-chloromethyl-2',7'-dichlorofluorescein. *Cytometry A* 2006, **69**:1184-1192.
10. **Riccardi C** and Nicoletti I. Analysis of apoptosis by propidium iodide staining and flow cytometry. *Nat. Protocols*, 2006, **1**:1458-1461,
11. **Pankiv S**, Clausen TH, Lamark T, Brech A, Bruun JA, Outzen H, et al. p62/SQSTM1 binds directly to Atg8/LC3 to facilitate degradation of ubiquitinated protein aggregates by autophagy. *J. Biol. Chem.* 2007, 282:24131-24145.
12. **Schöckel L**, Glasauer A, Basit F, Bitschar K, Truong H, Erdmann G, et al. Targeting mitochondrial complex I using BAY 87-2243 reduces melanoma tumor growth. *Cancer & metabolism* 2015, 3: 11.
13. **Śliwka L**, Wiktorska K, Suchocki P, Milczarek M, Mielczarek S, Lubelska K, Cierpiał T, Łyżwa P, Kiełbasiński P, Jaromin A, Flis A, Chilmonczyk Z. The Comparison of MTT and CVS Assays for the Assessment of Anticancer Agent Interactions. *PLoS One*, 2016, **11**:e0155772.
14. **Smiley ST**, Reers M, Mottola-Hartshorn C, Lin M, Chen A, Smith TW, et al. Intracellular heterogeneity in mitochondrial membrane potentials revealed by a J-aggregate-forming lipophilic cation JC-1. *Proc. Natl. Acad. Sci. USA*, 1991, **88**:3671-3675.
15. **Winther JR**, Thorpe C. Quantification of thiols and disulfides. *Biochim. Biophys. Acta.*, 2014, **1840**: 838-846.
